# Biocompatibility of Phosphorus Dendrimers and Their Antibacterial Properties as Potential Agents for Supporting Wound Healing

**DOI:** 10.1021/acs.molpharmaceut.4c01156

**Published:** 2025-01-11

**Authors:** Beata Bielska, Natalia Wrońska, Joanna Kołodziejczyk-Czepas, Serge Mignani, Jean-Pierre Majoral, Iveta Waczulikova, Katarzyna Lisowska, Maria Bryszewska, Katarzyna Miłowska

**Affiliations:** † Department of General Biophysics, Faculty of Biology and Environmental Protection, University of Lodz, 141/143 Pomorska St., 90-236 Lodz, Poland; ‡ Doctoral School of Exact and Natural Sciences, University of Lodz, 21/23 Jana Matejki Street, 90-237 Lodz, Poland; § Department of Industrial Microbiology and Biotechnology, Faculty of Biology and Environmental Protection, University of Lodz, 12/16 Banacha Street, 90-237 Lodz, Poland; ∥ Department of General Biochemistry, Faculty of Biology and Environmental Protection, University of Lodz, Pomorska 141/143, 90-236 Lodz, Poland; ⊥ CQM-Centro de Química da Madeira, Universidade da Madeira, Campus Universitário da Penteada, 9020-105 Funchal, Portugal; # Centre d’Etudes et de Recherche sur le Medicament de Normandie (CERMN), Université de Caen Normandie, Caen 14032, France; ∇ Laboratoire de Chimie de Coordination CNRS, 205 Route de Narbonne, Toulouse 31077, France; ○ Department of Nuclear Physics and Biophysics, Faculty of Mathematics, Physics and Informatics, Comenius University, Mlynska Dolina F1, 84248 Bratislava, Slovakia

**Keywords:** phosphorus dendrimers, wound healing, nanoparticles, skin, blood

## Abstract

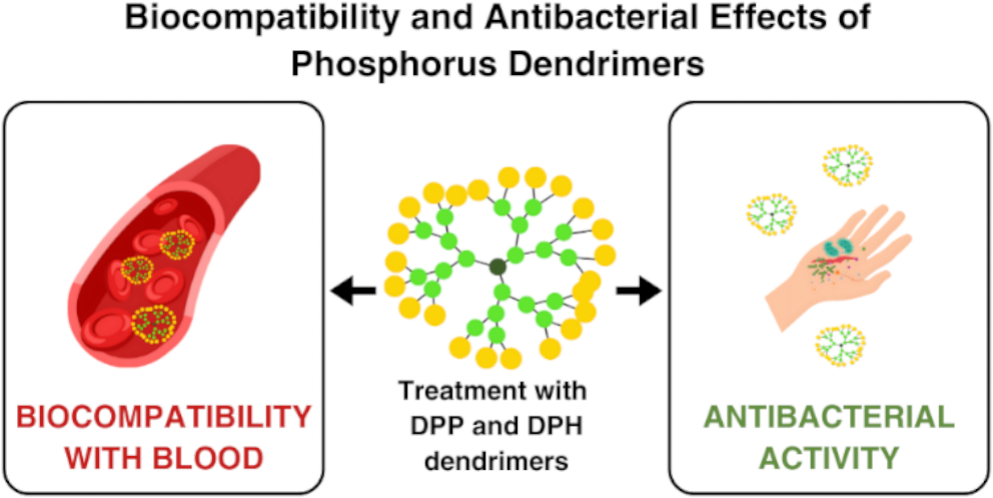

Dendrimers are a wide range of nanoparticles with desirable properties that can be used in many areas of medicine. However, little is known about their potential use in wound healing. This study examined the properties of phosphorus dendrimers that were built on a cyclotriphosphazene core and pyrrolidinium (DPP) or piperidinium (DPH) terminated groups, to be used as potential factors that support wound healing (*in vitro*). Therefore, the degree of toxicity of the tested compounds for human erythrocytes and the human fibroblast cell line (BJ) was determined, and it was found that at low concentrations, the tested compounds are compatible with blood. The influence of phosphorus dendrimers on plasma proteins (human serum albumin (HSA) and fibrinogen) was examined, with a lack of conformational changes in the structure of these proteins, suggesting that their physiological function was not disturbed. The effects on plasma coagulation cascade and fibrinolysis were also assessed, and it was found that phosphorus dendrimers in low concentrations are blood compatible and interfere neither with coagulation processes nor in clot breakdown. Skin injuries, especially chronic wounds, are also susceptible to infection; therefore, the antimicrobial potential of dendrimers was tested, and it was found that these dendrimers had antibacterial activity against both Gram-negative and Gram-positive bacteria. The highest activity of the tested compounds was found for higher applied concentrations.

## Introduction

Skin is the largest organ of the human body and is the first barrier separating organs and tissues from the external environment. Receptors are distributed on the skin through which stimuli from the environment are received. The skin also participates in the synthesis of vitamin D, which helps maintain homeostasis of many different biological systems, *e.g.*, accelerating wound healing.^1−4^ The treatment of skin wounds is a challenge of high significance, being associated with large financial outlays, so work has been underway to develop dressings that support wound healing.^5^

The healing process is a complex mechanism that involves a sequence of cellular events, leading to the formation of new healthy tissue. The sequence of events includes hemostasis, inflammation, proliferation, and remodeling.^6,7^ At each stage, the process of tissue repair can be disrupted and thus pathological changes can occur, causing health and ensuing financial problems.^8^

The lack of optimum performance in traditional dressings has led to the search for new promising materials that could overcome the shortcomings of current therapeutic agents. Natural and synthetic polymers have been widely used in medical preparations and scaffolds. Polymeric materials, due to several beneficial properties, have been used as a potential alternative in wound treatment, demonstrating their ability to accelerate wound healing.^9^ Dendrimers are widely studied nanoparticles and are three-dimensional (3-D), spherical molecules containing multiple end groups in their structure. Unlike traditional polymers, dendrimers have three distinct structural elements: a centralized core, a middle section consisting of repeating branches that make up each generation, and an outer shell defined by end functional groups.^10−12^ The specific structure of these compounds significantly affects their properties and is important for their use in medicine. Dendrimers may be drug carriers, but they also have therapeutic (*e.g.*, antiviral or antibacterial) properties or prevent the aggregation of peptides and proteins, which play a key role in neurodegenerative disorders.^13−24^

Studies suggest that dendrimers also have promising properties in the treatment of wound infection, reduction of excess inflammation in burn wounds, and relieving wound oxidative stress.^25−27^ In addition, dendrimers show antimicrobial activity against Gram-positive as well as Gram-negative bacterial strains. The mechanism of the antimicrobial action of dendrimers is mainly based on their ability to modify the permeability of the cell membrane of microorganisms.^28^ This suggests the possibility of using dendrimers as new tools in the treatment of chronic wounds to accelerate the healing process. This project aims to investigate whether new polycationic phosphorus dendrimers based on a cyclotriphosphazene core can be used in the wound healing process. Cyclotriphosphazene-based dendrimers were first synthesized in the early 1990s. Dendrimers whose core contains phosphorus are characterized by their stability at high temperatures. The thermal stability is not dependent on the generation of the dendrimer but is dependent on the internal structure and the type of end groups. They exhibit several diverse biological activities, acting as drug nanocarriers, and contrast agents in imaging or in diagnostics, but they are also active *per se*.^29−31^

The dendrimers have positive effects on the growth of neuronal cells, human monocytes, and natural killer (NK) cells.^32−34^ In addition, they have been tested as ocular drug delivery platforms (*in vivo*), transfection vectors, and imaging agents, including *in vivo* studies, and used for the production of sensitive DNA microarrays.^35−39^ Cationic phosphorus dendrimers (CPD) of the third (G3) and fourth (G4) generations can interact with charged and neutral lipid membranes and liposomes.^40,41^ Furthermore, they have the potential to fight against neurodegenerative diseases. It was discovered that CPD exhibits antiprion activity (also *in vivo*) and can inhibit aggregation of Aβ1–28 peptide and the MAPTau proteins involved in Alzheimer’s disease, as well as α- synuclein that is involved in Parkinson’s disease.^20,42,43^

The antibacterial activity of many dendrimers was studied in the past few years to examine their role as a novel drug, although not much for phosphorus dendrimers. Ciepluch and co-workers showed that dendrimers containing phosphorus in their structure have strong antimicrobial activity, inhibiting the growth of *Staphylococcus aureus* by 80% and reducing the growth of *Escherichia coli* by 50%.^44^ In addition, phosphorus dendrimers are used in combination with antibiotics, which have become less effective due to the development of antibiotic resistance. The coadministration of anionic phosphate dendrimers with levofloxacin reduces bacterial growth by 70%, while the use of the antibiotic alone results in only a 15% inhibition of growth.^45^ Synergistic effects have also been observed when phosphate dendrimers are combined with several different classes of antibiotics targeting drug-resistant mycobacterial pathogens. Phosphorus-based nanoparticles have shown the ability to enhance *in vitro* and *in vivo* the action of different antibiotic classes against one of the most drug-resistant pathogens, *Mycobacterium abscessus*.^46^ A recent study showed synergistic benefits of phosphorus dendrimers of the fourth generation with levofloxacin, resulting in a decreased antibiotic dose for the treatment of bacterial infection.^45^ Mignani et al. described an orally, bioavailable polycationic phosphorus dendrimer as a potential antimicrobial agent against *Mycobacterium tuberculosis*.^47^

This wide range of properties exhibited by phosphorus dendrimers prompted the evaluation of the impact of two selected dendrimers based on a cyclotriphosphazene core on the wound healing process. To achieve the goal mentioned above, the effect of dendrimers on blood cells (erythrocytes) and skin cells (fibroblasts) was assessed, as it is important that they did not show toxicity toward the cells with which they will be in direct contact when used for wound treatment. The interactions of dendrimers with selected blood plasma proteins involved in wound healing (albumin and fibrinogen) were also examined. Tests were also carried out on the antibacterial and hemostatic properties of dendrimers and their effect on fibrinolysis (*in vitro*). The results obtained determined whether the tested dendrimers could accelerate the process of platelet plug formation, stop bleeding, and protect against the action of microorganisms.

## Materials

The BJ human skin fibroblast cell line (CRL-2522) was purchased from the American Typer Culture Collection ATCC (Manassas, VA). Cell culture reagents, such as Dulbecco’s Modified Eagle Medium (DMEM), fetal bovine serum (FBS), and antibiotic (streptomycin and penicillin), were purchased from Gibco, ThermoFisher Scientific (Waltham, MA). Dimethyl sulfoxide (DMSO), 3-(4,5–2-yl)-2–5- diphenyltetrazolium bromide (MTT), phosphate-buffered saline (PBS) tablets, trypsin, and potassium ferrocyanide were purchased from Sigma-Aldrich (Saint Louis, MO). Reagents for the determination of clotting times (Dia-PT, Dia-TT, and DiaPTT) were purchased from Diagon (Budapest, Hungary). Reagents for clot formation and fibrinolysis (CFF) assays included the tissue-type plasminogen activator (Actilyse, Boehringer Ingelheim, Ingelheim am Rhein, Germany) and thrombin (Sigma-Aldrich, Saint Louis, MO). Mueller–Hinton broth was obtained from Becton Dickinson (Warsaw, Poland).

The proteins used in the study were human serum albumin (HSA) and fibrinogen and were purchased from Sigma-Aldrich (USA).

Fresh human blood plasma for hemostatic assays derived from buffy coats (from healthy donors) was purchased from the Regional Centre of Blood Donation and Blood Treatment in Lodz (Poland). The buffy coat units were anonymized before the purchase, ensuring that the studies did not involve the acquisition and processing of any personal data. All experiments involving blood-derived cells or plasma were approved by the committee on the Ethics of Research at the University of Lodz.

**Figure 1:**
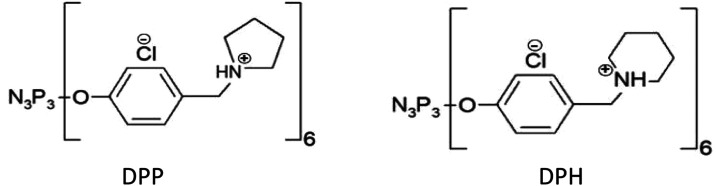
Chemical structure of phosphorus dendrimers.

Generation zero polycationic phosphorus dendrimers were synthesized at the CNRS Coordination Chemistry Laboratory in France. The synthesis and characterization of DPP and DPH dendrimers were described in previous work.^47^ The chemical structure of the phosphorus dendrimers is shown in Figure 1, and selected dendrimer parameters are shown in Table 1. The dendrimers were dissolved in 10 mM phosphate buffer at pH 7.4.

**Table 1:** Chemical Characteristics of the Tested Dendrimers

dendrimer	core	terminal groups	molecular weight [g/mol]
DPP	cyclotriphosphazene (N_3_P_3_)	pyrrolidinium	1411,12
DPH	cyclotriphosphazene (N_3_P_3_)	piperidinium	1495,29

## Methods

### Cell Culture

Human skin fibroblast (BJ) cells were cultured as a monolayer in DMEM growth medium with GlutaMAX (l-glutamine) supplemented with 10% fetal bovine serum (FBS) and 1% penicillin and streptomycin solution. Cultures were incubated at 37 °C in an atmosphere of 95% air and 5% CO_2_ at 100% relative humidity. Cell lines were divided into subcultures every 2 days and maintained in a logarithmic growth phase by regular passaging into new culture bottles once cells reached ∼80% confluence. Monolayers were washed sequentially with isotonic saline solution (0.9%), and then, the cells were trypsinized by adding 0.25% trypsin. Cells were incubated for 3–5 min in an incubator, and the degree of detachment was monitored under a microscope. After detachment, the DMEM and GlutaMAX culture medium were added to the BJ cells. Cell viability was assessed at each passage using trypan blue, which selectively stains dead cells with damaged cell membranes blue. A small amount of the cell suspension (10 μL) was mixed 1:1 with trypan blue and applied to a slide. When measured on a Countess cell counter (Korea), the total cell density and the number of dead and live cells in 1 mL of the suspension were determined.

### MTT Test

The cytotoxicity of the phosphorus dendrimers was assessed by using the MTT assay. BJ cells were seeded into 96-well plates at a density of 1 × 10^5^ cells/mL, and then, the plates were incubated for 24 h in an incubator at 37 °C and 5% CO_2_ atmosphere. After 24 h, the culture medium was removed, and the cells were washed with PBS. Then, 3-(4,5-dimethylthiazol-2-yl)-2,5-diphenyltetrazolium bromide (MTT) was added at a concentration of 0.5 mg/mL, and the probes were incubated for 3 h in an incubator (37 °C, 5% CO_2_ atmosphere). After 3 h, the excess MTT was removed from the plates, and DMSO was added to dissolve the formed formazan crystals. Finally, absorbance was measured for each well at λ = 570 and 720 nm on a BioTek Synergy HTX Reader spectrophotometer (Winooski, Vermont). Using the spectrophotometer software, the difference between the measurements at the indicated wavelengths was obtained in the form of an absorbance delta (Δ*A*). The percentage of cell viability was determined by the percentage ratio of absorbance of the sample containing the compound to the control without the compound, according to the equation:
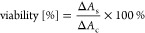
where:

Δ*A*_s_ is the absorbance of the sample after the addition of dendrimers

Δ*A*_c_ is the absorbance of the control (untreated cells)

### Hemolysis

The evaluation of the effect of dendrimers on the degree of hemolysis was performed on red blood cells isolated from the human leukocyte-platelet buffy coat by centrifugation at 3000 rpm (10 min, 4 °C) and purified by 3 washing cycles with PBS (buffered saline; pH = 7.4). Isolated red blood cells (RBCs) were resuspended in PBS to a final hematocrit (HTC) of 10%, which was then treated with dendrimer solution at concentrations of 1–50 μM and incubated at 37 °C for 24 h. Erythrocytes in PBS (without phosphorus dendrimer) were used as the control, and erythrocytes in water were used as 100% hemolysis. After incubation, samples were centrifuged (3000 rpm, 10 min) and absorbance was measured at λ = 540 nm on a BioTek Synergy HTX Reader spectrophotometer (Winooski, Vermont) to calculate the percentage of hemolysis according to the following equation:
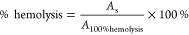
where

*A*_s_ is the sample absorbance

*A*_100%hemolysis_ is the absorbance of erythrocytes in water (100% hemolysis)

### Methemoglobin

The degree of oxidation of hemoglobin to methemoglobin under the influence of phosphorus dendrimers was assessed spectrophotometrically. The erythrocyte suspension was treated with phosphorus dendrimers at concentrations ranging from 1 to 50 μM and incubated at 37 °C for 24 h. The negative control composed of erythrocytes in PBS (without the phosphate dendrimer) and the positive control (100% methemoglobin) was a sample treated with potassium ferrocyanide. After the incubation time, distilled water was added to release hemoglobin from the erythrocytes. Samples were centrifuged (5000 rpm, 15 min), and absorbance was measured at λ = 630 and 700 nm on a BioTek Synergy HTX Reader spectrophotometer (Winooski, Vermont) to calculate the percentage of methemoglobin according to the following equation:
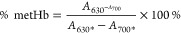
where:

*A*_630_ is the absorbance of control and sample with dendrimer at 630 nm,

*A*_700_ is the absorbance of control and sample with dendrimer at 730 nm,

*A*_630*_ is the absorbance of control and sample with dendrimers treated with potassium ferrocyanide (100% metHb) at 630 nm,

*A*_700*_ is the absorbance of control and sample with dendrimers treated with potassium ferrocyanide (100% metHb) at 700 nm.

### Fluorescence Measurements

Fluorescence spectroscopy is a technique used to study and analyze the structure of proteins. Interactions between plasma proteins and dendrimers can be observed by changes in the fluorescence intensity. Human serum albumin (HSA) and fibrinogen (FIB) contain tryptophan residues in their structure responsible for triggering the fluorescence phenomenon upon excitation with the appropriate wavelength, thus making it possible to study changes in protein conformation.

Determination of intrinsic tryptophan fluorescence in proteins (human serum albumin and fibrinogen) was carried out on a PerkinElmer LS-50B spectrofluorometer (Shelton, Connecticut) at an excitation wavelength of 280 nm, emission wavelengths of 305–450 nm, slit widths of 10 and 10 for human serum albumin, an excitation wavelength of 280 nm, emission wavelengths of 295–450 nm, and slit widths of 3 and 3 for fibrinogen. Measurements were carried out at 37 °C in a quartz cuvette with an optical path length of 5 mm. Increasing concentrations of dendrimers were added to the cuvette containing the protein (human serum albumin at a concentration of 5 μM or fibrinogen at a concentration of 0.5 μM) to obtain a dendrimer–protein molar ratio of 1:1 to 1:20 and the fluorescence of the tryptophan residues was measured.

Before the tryptophan fluorescence was measured, the dendrimers themselves were examined to see if they showed fluorescence in the wavelength range under investigation.

### Circular Dichroism

Circular dichroism (CD) spectroscopy is used to determine the properties of optically active compounds that exhibit the ability of a substance to rotate the plane of polarization of a beam of light that is passed through it (Miles et al. 2021). Analysis of CD spectra in the far ultraviolet (UV) region gives an estimation of the secondary structure of the tested protein. CD spectra (195–260 nm) were performed for the proteins (HSA and fibrinogen) in the presence of phosphorus dendrimers using a J-815 JASCO CD spectropolarimeter. The concentration of the proteins in the sample was 0.5 μM, while the dendrimers used ranged from 0.5 to 10 μM. Changes in the secondary structure of the proteins in the presence of dendrimers were studied at 37 °C.

### Prothrombin (PT) and Thrombin (TT) Time Measurement

Prothrombin time (PT) provided information for the efficiency of the extrinsic blood coagulation pathway (the tissue factor-dependent pathway of blood plasma coagulation cascade) and included the activation of factor VII and activity of components of the common pathway (mainly factor V, X, thrombin, and fibrinogen).

Thrombin time (TT) is assessed as the amount of time required to convert blood plasma fibrinogen into insoluble fibrin. This parameter provides information on the executive phase of the plasma coagulation cascade, *i.e.*, the thrombin-catalyzed fibrinogen polymerization and formation of the fibrin clot.

PT and TT were determined coagulometrically using a K-3002 analyzer (KSELMED, Grudziadz, Poland) according to the protocol provided by the manufacturer. Blood was centrifuged for 15 min at 4200 rpm at 25 °C. Samples containing dendrimers in 5 μL volumes at concentrations of 1–50 μM were added to 495 μL of plasma. The samples were then incubated with plasma for 15 min at 37 °C. After incubation, 50 μL of dendrimer-treated plasma and 100 μL of the Dia-PT reagent (DIAGON, Budapest, Hungary; a commercial preparation was dissolved in 2 mL of deionized water) were added to the cuvettes, and the plasma clotting time (PT) was measured. In order to measure the TT time after a 15 min preincubation, 100 μL of plasma and 100 μL of Dia-TT reagent, *i.e.*, thrombin (dissolved in 3 mL of deionized water), were added to the cuvette.

### Activated Partial Thromboplastin Time (aPTT)

The activated partial thromboplastin time (aPTT) provided information on the efficiency of the intrinsic blood coagulation pathway (the contact factors-dependent pathway). This test is based on determining the time of clot formation as induced by the presence of phospholipids and contact-phase activators (*e.g.*, kaolin), with a cofactor activity of calcium ions. APTT depends on the activity of coagulation factors in the intrinsic pathway (mainly XII and XI) and the components of the common pathway (*i.e.*, the factor IX, X, VIII, thrombin, and fibrinogen). Lyophilized DiaPTT reagent was reconstituted in 4 mL of deionized water. Measuring cuvettes were placed in a thermostat of a K-3002 coagulometer (KSELMED, Grudziadz, Poland), and 50 μL of plasma (control or preincubated for 15 min with the examined dendrimers) was added into the cuvette. Dia-PPT reagent was then added to the cuvette with plasma. After a 3 min incubation at 37 °C, 50 μL of 0.025 M CaCl_2_ was added to start the measurement of plasma clotting.

### Clot Formation and Fibrinolysis (CFF) Assay

The CFF assay is a two-step test for kinetic analysis of both the blood plasma clotting process and its fibrinolytic capacity based on blood plasma turbidity changes. In this method, the thrombin-induced plasma polymerization and fibrin clot stabilization were followed by tissue-type plasminogen activator (t-PA)-induced fibrin degradation. The assay procedure was conducted according to the previously established protocol.^48^ Absorbance was measured continuously at a wavelength of 360 nm every 10 s for 30 min at 37 °C, using a SpectrostarNano microplate spectrophotometer (BMG LabTech, Ortenberg, Germany). The reagent mixture was based on 0.05 M tris-buffered saline (pH 7.4) and contained the thrombin (at a concentration of 0.75 U/mL), the t-PA (at a concentration of 0.264 ng/mL), and 7.5 mM CaCl_2_. As a reference, the anticoagulant drug argatroban (a thrombin inhibitor) was used.

### Antibacterial Activity

The antibacterial activity of DPP and DPH dendrimers was evaluated using the microdilution method for aerobic bacterial strains, according to the CLSI documents M07 (11th Edition).^49^ The antibacterial activity was tested against the following aerobic bacteria (*S. aureus* ATCC 6538, *S. aureus* ATCC 25923, *S. aureus* ATCC 700699, *Streptococcus pyogenes* ATCC 19615, *Proteus hauseri* ATCC 15442, *Pseudomonas aeruginosa* ATCC 27853, and *E. coli* ATCC 25922). Growth of the bacterial strains was treated either with the DPP or DPH dendrimers or left untreated and was evaluated in 96-well microtiter plates in Mueller–Hinton broth. The antibacterial potential of tested compounds was for a concentration range of 0.5–10 μM. The dendrimers were dissolved in phosphate buffer, and then, the stock solutions were diluted in a Mueller–Hinton medium. An inoculum of bacteria grown in the Mueller–Hinton broth was added to each well to achieve a final density of 5 × 10^5^ CFU/mL. The microtiter plates were then incubated for 24 h at 37 °C. After incubation, the optical density of all of the samples was measured at λ = 630 nm, using a microplate reader (Multiskan FC Microplate Photometer, ThermoFisher Scientific, Pudong, Shanghai, China). The growth of the tested bacterial strains was calculated as a percentage of the control (bacteria incubated in the medium), based on the values of optical density from 6 experiments (*n* = 6).

### Statistical Analysis

Results were presented as mean values with standard deviations (SD) and were processed using the Statistica software. The normality of the distribution was assessed using the Shapiro-Wilk parametric test. In the case of normally distributed data, parametric tests based on analysis of variance (ANOVA) were applied; otherwise, the nonparametric alternative (the Kruskal–Wallis test) was used. Statistical significance was assumed for *p* < 0.05.

## Results

### Cytotoxicity (MTT)

**Figure 2:**
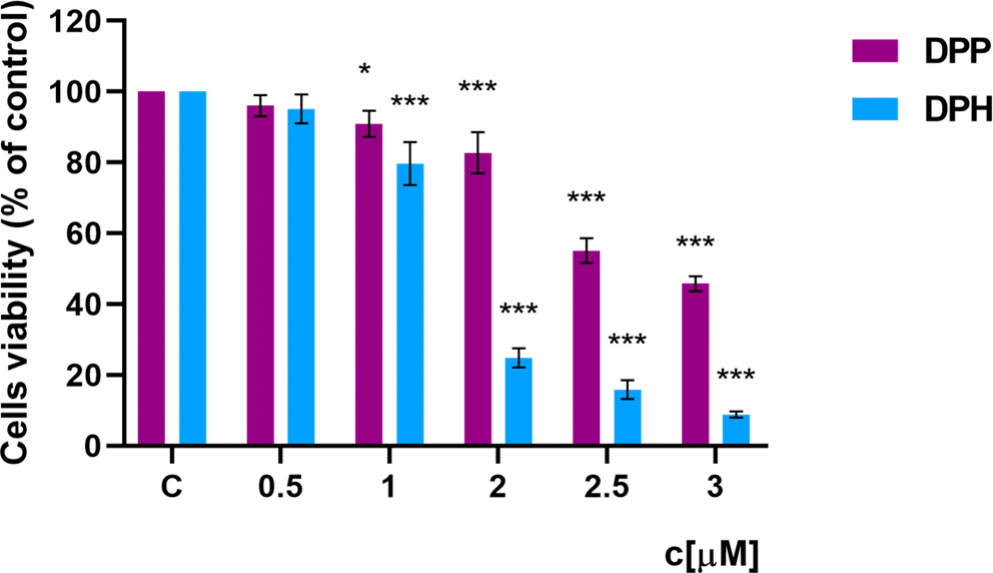
Viability of BJ cells treated with dendrimers: DPP and DPH for 24 h (*n* = 6, * *p < 0.05* *** *p < 0.001* compared to the control).

To determine the cytotoxicity of the tested compounds on human skin fibroblasts (BJs), we performed an MTT assay was performed. The cytotoxicity of the dendrimers was assessed by cell viability after 24 h of incubation (Figure 2). The percentage of viable cells was calculated by reference to the control cells not treated with the compound, and the viability was considered to be 100%. Dendrimers DPP and DPH significantly reduced the BJ cell viability in the range of concentrations used. Their toxicity increased progressively with an increasing compound concentration. Both dendrimers caused a statistically significant reduction in cell viability starting from a concentration of 1 μM. In the case of the DPP dendrimer, a sudden reduction in cell viability by 50% was observed at a concentration of 2.5 μM. However, in the case of the DPH dendrimer, a dynamic decrease in viability was noticeable with increasing concentration. A large decrease in cell number was observed at a concentration of 2 μM, at which the viability was only 25%. At the highest concentration used, the compound caused almost complete inhibition of the cellular metabolic activity. For both dendrimers, the IC50 was defined. The IC50 value for DPP was higher than for DPD, 2,811 μM, and 1,475 μM, respectively. The DPP dendrimer was less toxic to skin cells than the DPH dendrimer.

### Hemolytic Activity

**Figure 3:**
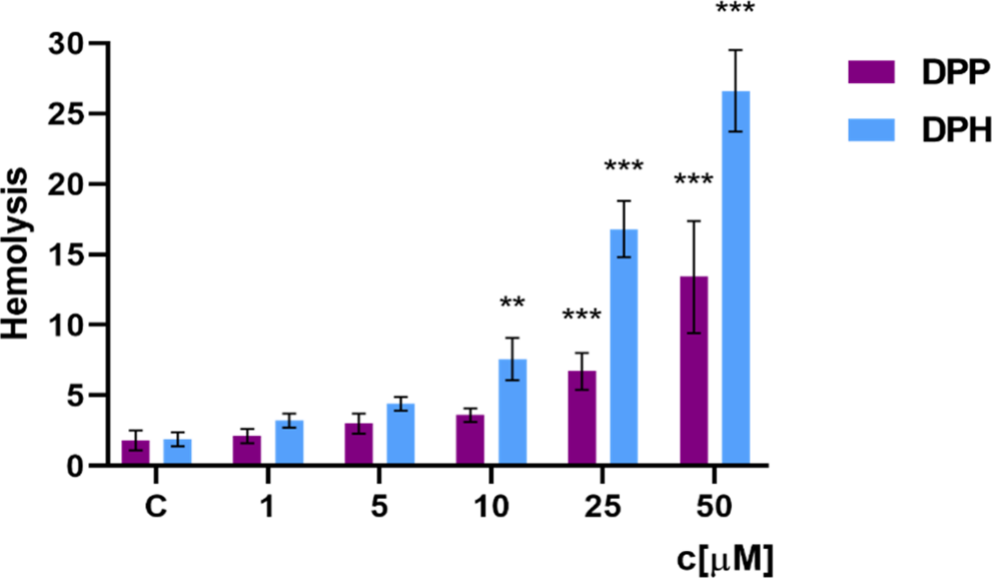
Hemolysis of erythrocytes incubated with dendrimers: DPP and DPH for 24 h (*n* = 6, ** *p < 0.01* *** *p <* 0.001 compared to the control).

Hemolysis of human erythrocytes was determined by measuring the hemoglobin content of the suspension after incubation with dendrimers DPP and DPH for 24 h (Figure 3). It was observed that both the DPP and DPH dendrimers-induced hemolysis. The degree of hemolysis depended on the concentration and type of dendrimer. For the DPP dendrimer, a statistically significant increase in the degree of hemolysis was observed from the concentration of 25 μM, and the highest degree of hemolysis was 13.4% for a concentration of 50 μM. For the DPP dendrimer, statistically significant changes were observed from a concentration of 10 μM. The DPH dendrimer at a concentration of 50 μM induced a 2-fold higher degree of hemolysis than DPP. The results showed that the dendrimer DPH was more toxic to erythrocytes than DPP.

### Methemoglobin

**Figure 4:**
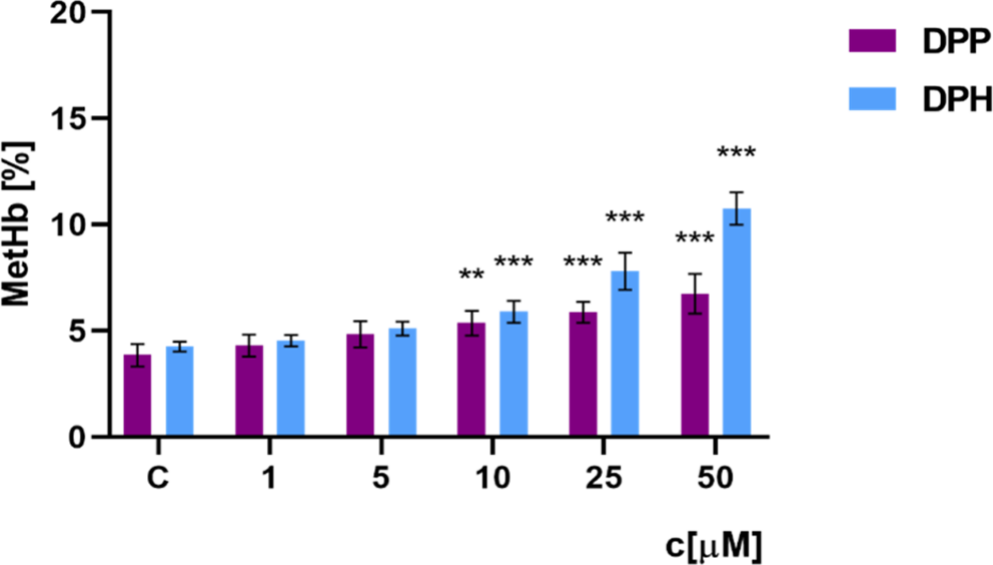
Percentage of methemoglobin in erythrocytes incubated with dendrimers DPP and DPH for 24 h (*n* = 6, ** p < 0.01 *** p < 0.001 compared to the control).

The tested dendrimers caused a slight oxidation of hemoglobin after 24 h of incubation with erythrocytes (Figure 4). The percentage of methemoglobin after 24 h in the control was 4%, while the amount of methemoglobin in erythrocytes gradually increased with the increasing dendrimer concentration. These changes are statistically significant from a concentration of 10 μM onward for both dendrimers tested. For DPP, the highest methemoglobin content was only 6.7% for a concentration of 50 μM, while for the DPH, it was 10.8% for the same concentration. Dendrimer DPH oxidized hemoglobin to a greater extent in erythrocytes than DPP did.

### Quenching Protein Fluorescence

**Figure 5:**
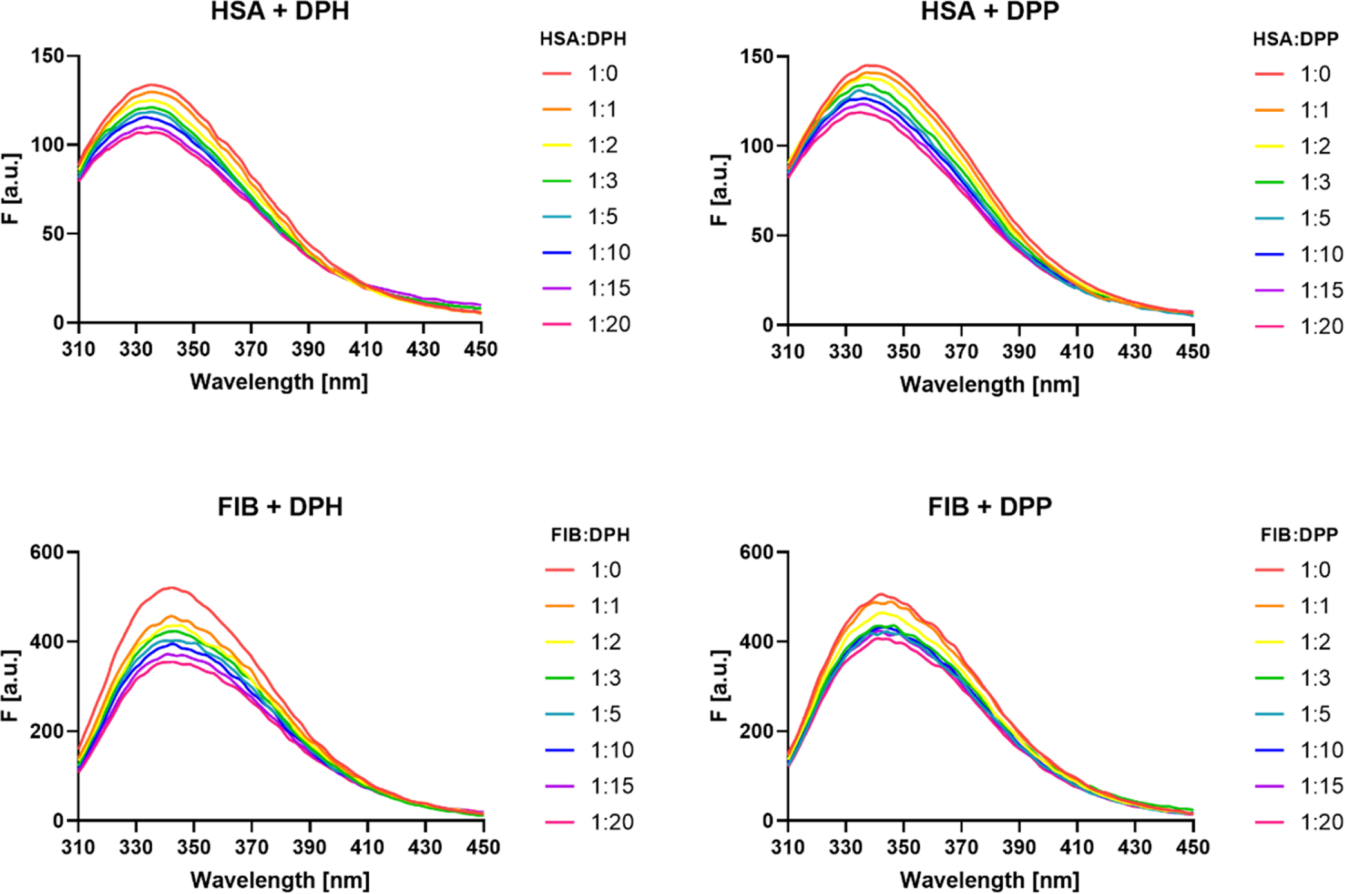
Fluorescence spectra of fibrinogen (FIB) (*c* = 0.5 μM) and human serum albumin (HSA) (*c* = 5 μM) after addition of dendrimers DPP and DPH at respective molar ratios.

The fluorescence spectra of fibrinogen and human serum albumin in the presence of dendrimers are shown in Figure 5. When dendrimers were added to proteins, a decrease in the fluorescence intensity of the tryptophan residues within the protein was observed. It can therefore be concluded that the 2 polycationic phosphorus dendrimers of a low generation based on a cyclotriphosphazene core were capable of quenching the fluorescence of tryptophan residues in fibrinogen and human serum albumin to a small extent. Additionally, after adding dendrimers to HSA, a shift of the emission maximum toward shorter wavelengths was observed. In contrast, there was no significant shift in the maximum emission wavelengths in the fibrinogen spectra.

### Secondary Structure of Human Serum Albumin and Fibrinogen

**Figure 6:**
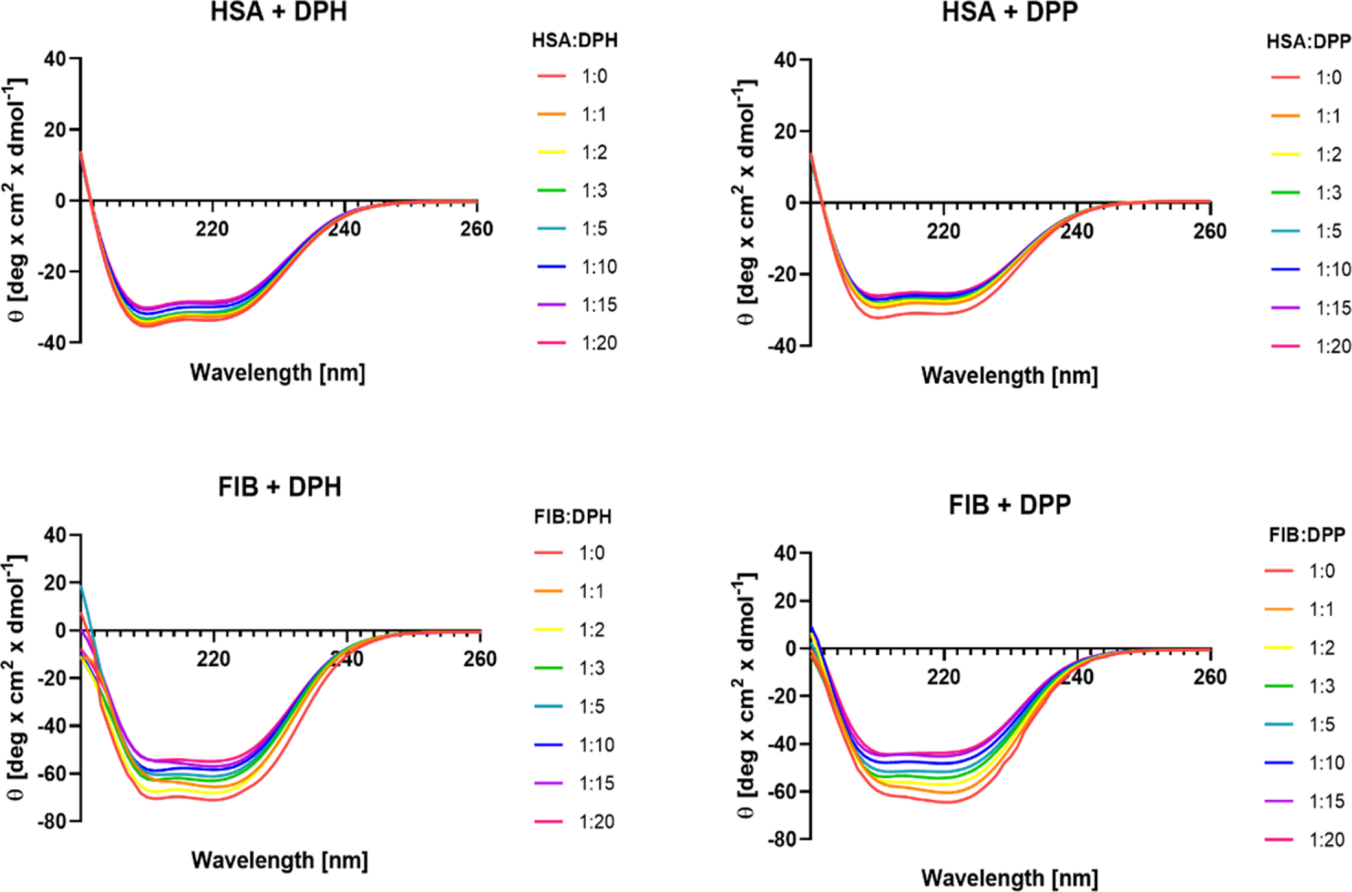
CD spectra of fibrinogen (FIB) (*c* = 0.5 μM) and human serum albumin (HSA) (*c* = 0.5 μM) in the presence of dendrimers: DPP and DPH at respective molar ratios (*n* = 3).

Using the circular dichroism method, we determined the effect of phosphorus dendrimers on the secondary structure of plasma proteins was determined. The spectra of fibrinogen and human serum albumin alone and with dendrimers are presented in Figure 6. The dendrimers DPP and DPH had no optical activity in the wavelength range and concentration tested. Both HSA and fibrinogen are characterized by the presence of α-helixes in their structure with 2 minima around 210 and 220 nm. After the addition of dendrimers in the tested concentration range, there was a gradual decrease in the intensity of the negative signal in the spectra. In both cases, there was a similar level of reduction of the negative signal intensity. However, no significant changes in the shapes of the spectra of the tested proteins were observed. The results indicate that dendrimers interacted with proteins but did not significantly change the secondary structure of fibrinogen or HSA.

### Assessment of Hemostatic Properties

To determine the effect of the phosphorus dendrimers on the activation of intrinsic and extrinsic pathways of the plasma coagulation system, commonly used diagnostic biomarkers, namely, blood coagulation times, were measured. The prothrombin time (PT), thrombin time (TT), and activated partial thromboplastin time (aPTT) were determined after 15 min of incubation with dendrimers. The results indicated a tendency toward the blood clotting time to be extended slightly with an increase in the concentration of dendrimers when compared to the control plasma, where the clotting time was 15.8 s. At the highest concentration of both tested dendrimers (50 μM), the clotting time increased to approximately 17.5 s; however, these changes are not statistically significant (Table 2).

**Table 2:** Effect of Dendrimers DPP and DPH on the Hemostatic Properties of Human Plasma: Prothrombin Time Parameters; Thrombin Time Parameters; and Partial Thromboplastin Time Parameters After Activation (*n* = 8, ** p < 0.01 *** p < 0.001 relative to the control)

type of sample	concentration [μM]	PT	TT	APTT
DPP	control	15.8 ± 1.78s	20.6 ± 0.67s	46.5 ± 3s
	1	15.9 ± 1.70s	20.8 ± 0.37s	48.7 ± 3.3s
	5	16.3 ± 1,78s	20.8 ± 0.56s	51.9 ± 5.7s
	10	16.5 ± 1,70s	20.7 ± 0.40s	62.6 ± 6.3s**
	25	17.0 ± 1,73s	20.6 ± 0.24s	92.3 ± 14.8s***
	50	17.4 ± 1,79s	20.6 ± 0.59s	total inhibition
DPH	control	15.8 ± 1,78s	20.6 ± 0.67s	46.5 ± 3s
	1	15.9 ± 1,67s	20.6 ± 0.39s	47.4 ± 3.2s
	5	16.2 ± 1,87s	20.4 ± 0.32s	48.6 ± 4s
	10	16.4 ± 1,69s	20.7 ± 0.43s	50.4 ± 5.5s
	25	16.9 ± 1,77s	20.4 ± 0.60s	62.1 ± 8.9s**
	50	17.5 ± 1,81s	20.7 ± 0.26s	81.0 ± 15.4s***

For the measurement of thrombin time, no statistically significant changes were observed after incubation of plasma with the 2 phosphorus dendrimers. Neither DPP nor the DPH dendrimer affected the time at which soluble fibrinogen was converted to insoluble fibrin (Table 2).

To determine the effect of the tested dendrimers on the intrinsic blood coagulation pathway, the partial thromboplastin time after activation was measured (Table 2). A prolongation of the blood clotting time was observed compared to the control plasma sample, which was 46.5 ± 3 s. In the case of the DPP dendrimer at the highest concentration of 50 μM, there was 100% inhibition of the clotting process. At the same time, the DPH dendrimer at a concentration of 50 μM extended the clotting time to 81.0 ± 15.4 s. At other concentrations, the DPH dendrimer slightly prolonged the blood clotting time compared to the control (by a maximum of 16 s). The changes were statistically significant for concentrations of 25 and 50 μM. A much greater difference was observed for the DPP dendrimer, where the changes in blood clotting time were significant starting from a concentration of 10 μM and the aPTT time increasing to 92 s for a concentration of 25 μM. This analysis led to the conclusion that the DPP dendrimer significantly affected the intrinsic blood coagulation pathway more than the DPH dendrimer did.

### Clot Formation and Fibrinolysis (CFF) Assay

**Figure 7:**
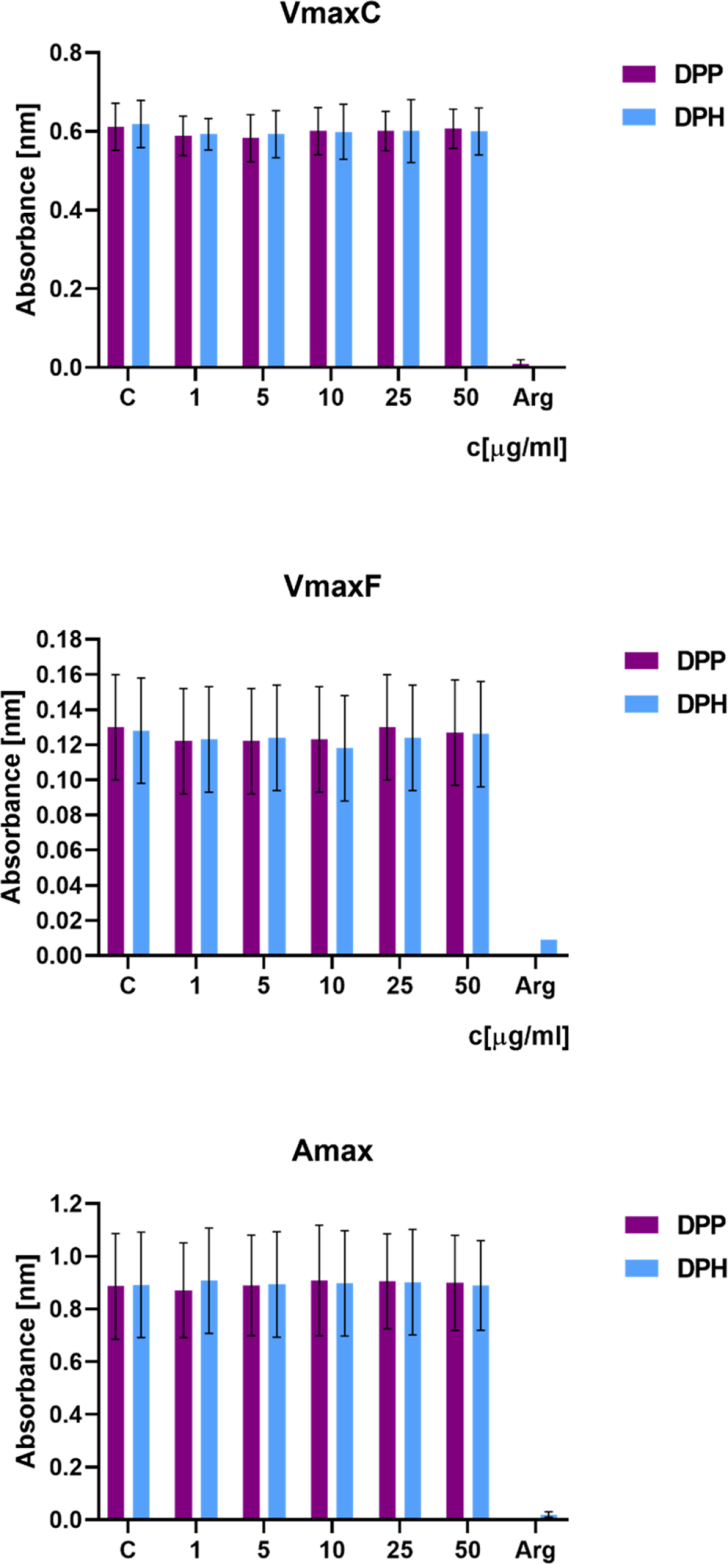
Effect of the tested DPP and DPH dendrimers on the hemostatic properties of human blood plasma (*n* = 12).

The CFF assay enabled analysis of both the blood coagulation (the thrombin-catalyzed fibrinogen polymerization) and fibrinolysis efficiency in the presence of the examined dendrimers. The assay provided 3 main parameters: *V*_maxC_ - the maximal velocity of fibrinogen polymerization (data related to the efficiency of blood plasma coagulation process), *A*_max_ (the maximal absorbance of the clotted plasma)—information on the fibrin clot stabilization and its thickness, and *V*_maxF_ (the maximal velocity of clot lysis)—a parameter reflecting the fibrinolysis efficiency (Figure 7).

Measurements of the *V*_maxC_ parameter indicated that the examined dendrimers did not influence the kinetics of the thrombin-induced fibrin polymerization, this being the executive phase of blood coagulation. These results were consistent with data from the thrombin time measurements. In addition, the examined substances had no effect on the fibrin clot structure (the *A*_max_ parameter). DPP and DPH dendrimers did not affect the fibrinolysis rate (the *V*_maxF_ parameter).

### Antibacterial Activity

**Figure 8:**
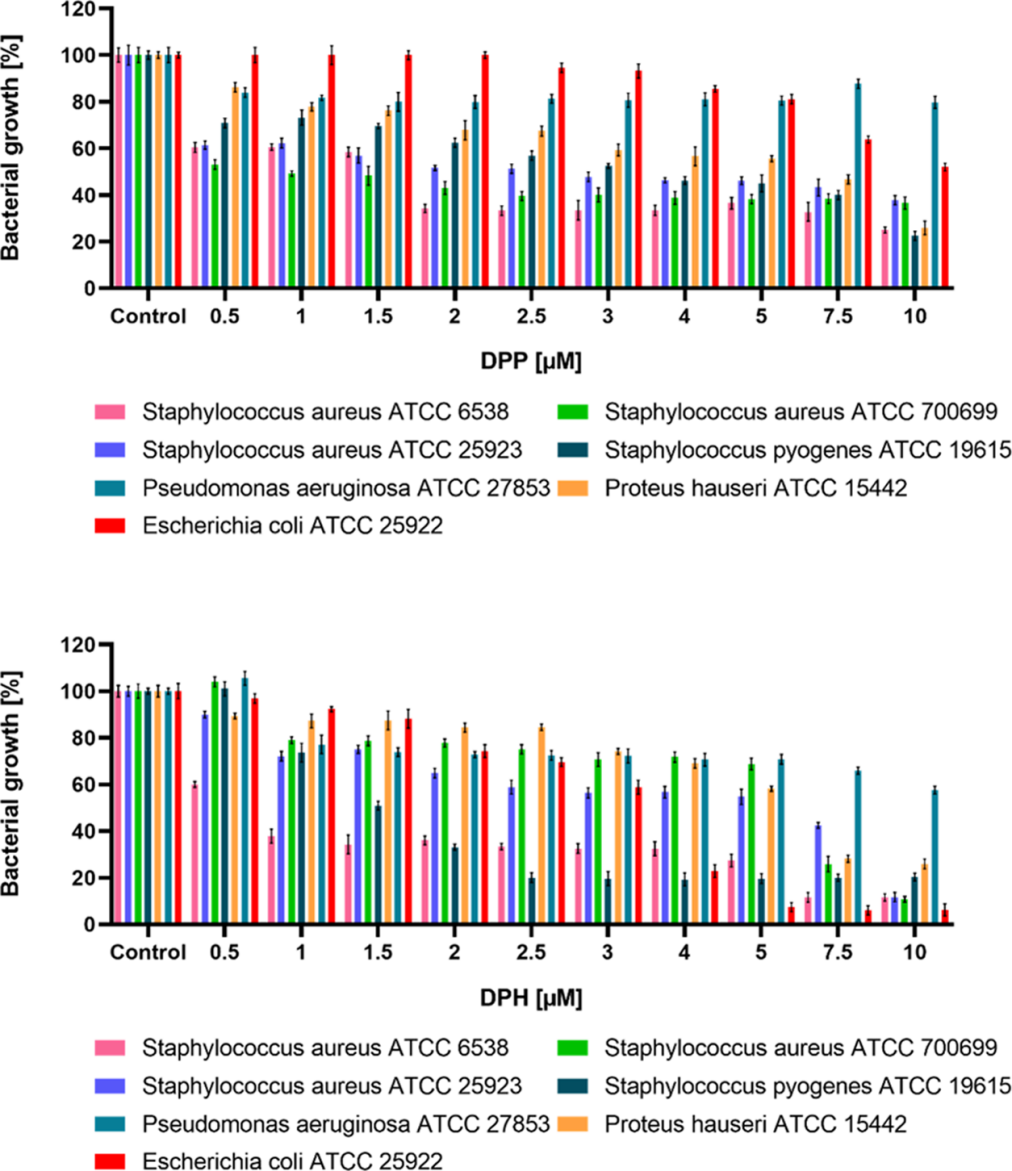
Growth of tested Gram-positive and Gram-negative bacteria in the presence of DPP and DPH dendrimers. Statistical differences occur for all bacteria between all tested concentrations of DPP and the control (expect *E. coli* up to 4 μM of DPP). Statistical differences occur for all bacteria between all tested concentrations of DPH and control (expect *S. aureus* ATCC 700699, *S. pyogenes* ATCC 19615, *P. aeruginosa* ATCC 27853, and *E. coli* for *c* = 0.5 μM of DPH).

The antimicrobial activity of 2 phosphorus dendrimers was evaluated against several human pathogens: *S. aureus* ATCC 6538, *S. aureus* ATCC 25923, *S. aureus* ATCC 700699, *S. pyogenes* ATCC 19615, *P. hauseri* ATCC 15442, *P. aeruginosa* ATCC 27853, and *E. coli* ATCC 25922. The results showed that dendrimers exhibited different activities against the tested bacteria. DPH dendrimers showed the greatest antimicrobial activity against Gram-positive strains: *S. aureus* ATCC 6538 and *S. pyogenes* ATCC 19615. Significant inhibition of the growth of *S. aureus* ATCC 6538 was observed in the culture incubated with the addition of DPH dendrimers at a concentration of 1 μM (Figure 8). At higher concentrations (below 2 μM), the DPH dendrimer caused a 65–80% growth inhibition of *S. aureus* ATCC 6538 and *S. pyogenes* ATCC 19615. Among Gram-positive strains, dendrimer DPH had the weakest effect on *staphylococci* strains (ATCC 25923, ATCC 700699). *E. coli* was the most sensitive Gram-negative microorganism, with intense growth inhibition observed at a concentration of 4 μM of dendrimer. In the case of the DPP dendrimer, bacterial growth inhibition was observed even at the lowest concentration of the compound. It showed the highest antimicrobial activity against *staphylococcal* strains (Figure 8). At a concentration of 2 μM, approximately 50–66% growth inhibition was observed. Gram-negative pathogens exhibited a relatively good tolerance to the DPP dendrimers. The most sensitive Gram-negative strain was *P. hauseri*.

## Discussion

Wound healing is a complex process influenced by many factors, including growth factors, cytokines, different cell types, and their interactions.^48,50^ One of the main problems during healing is the possibility of rapid colonisation of the wound by microorganisms and fungi, which delays the regeneration process.^51^ In the search for new therapies, researchers have turned their attention to dendrimers, which have unique anti-inflammatory, antifungal, and antimicrobial properties.^15,27,52,53^

Phosphorus dendrimers, particularly third- and fourth-generation cationic dendrimers, have shown the ability to interfere with β-amyloid and MAPTau protein aggregation in Alzheimer’s disease, suggesting their potential use in neurodegenerative therapy.^20,43,54^ However, their toxicity, which is dependent on positive charge and generation, is a significant limitation.^52,55^ Nevertheless, research into their use in the treatment of skin damage continues due to their promising therapeutic properties.

We studied whether the new phosphorus dendrimers of low generation, which are built on a cyclotriphosphazene core and terminated with either pyrrolidinium (DPP) or piperidinium groups (DPH), have properties supporting the wound healing process. It is necessary that any new compound or medicinal product intended for human use must also be compatible with blood components. The speed of blood clotting also plays an important role in the early stages of healing and fighting an infection. Therefore, it is important to check whether phosphorus dendrimers have antibacterial properties and whether they can accelerate the formation of a platelet plug, which will stop bleeding and protect the wound against the development of an infection.

Hemotoxicity tests confirmed the compatibility of the tested compounds at low concentrations with human blood. The 2 considered phosphorus dendrimers DPP and DPH at low concentrations did not damage the erythrocyte membrane and did not oxidize hemoglobin. The degree of hemolysis and the level of methemoglobin after 24 h of incubation with dendrimers were similar to those in the control sample that was not treated with the compounds. However, dendrimers at higher concentrations significantly affected these parameters in a dose-dependent manner. For the highest concentration (50 μM), a degree of hemolysis of 13% was observed for DPP and 2 times higher for DPH. The level of methemoglobin was also higher in the DPH-treated erythrocytes.

The viability of cells treated with these dendrimers was assessed using the MTT assay by evaluating the mitochondrial dehydrogenase activity. DPP and DPH dendrimers did not show any significant cytotoxicity toward human skin fibroblasts when applied up to a concentration of 1 μM, while dendrimers at higher concentrations caused a significant decrease in the viability of the tested cells. The decrease in cell viability depended on the concentration of dendrimers and their type. The results indicated that DPH was more toxic to skin fibroblasts than DPP. Similar strong cytotoxic effects were observed for the second and third generation of cationic phosphorus dendrimers (CPD), the activity of which was tested on mouse embryonic hippocampal cells (mHippoE-18) and neuroblastoma (N2a) cells. A significant decrease in cell viability was observed at concentrations above 1 μM for the third generation CPD dendrimer, which was correlated with the distribution in cellular activities, such as massive generation of ROS.^15^

Based on the results evaluating the toxicity of the dendrimers, the DPH dendrimer was found to be more toxic against both erythrocytes and skin cells. Both dendrimers were of the same generation (generation 0) and had the same number of positive charges. They differed only in the structures of the surface groups. The DPP dendrimer had 6 pyrrolidinium end groups, and DPH had 6 terminal piperidinium groups. The observed differences in toxicity of these dendrimers were therefore due to the nature of the surface groups, and it was assumed that this could be due to greater flexibility or accessibility to the positive charge on the azacyclohexane group.

Shcharbin et al. reviewed the biodistribution, toxicity, and pharmacokinetics of various dendrimers in animals. The data showed that almost all low- and intermediate-generation dendrimers were nontoxic *in vivo*, despite showing some cytotoxic effects *in vitro*. Only high generations of unmodified cationic dendrimers at high doses exhibited some toxicity *in vivo*. Any modification of the dendrimers (e.g., pegylation, coupling to poly(ethylene oxide), neutralization of the surface charge by biotin, sugar, or lipid attachment) led to reduced toxicity compared to unmodified dendrimers. This means that phosphorus dendrimers, despite some toxicity *in vitro*, can still be used for *in vivo* applications.^56^

In this study, the effect of phosphorus-containing dendrimers on the structure of 2 selected blood plasma proteins (HSA and fibrinogen) was assessed. These proteins were chosen because HSA catabolism in peripheral tissues increases after injury, while leading to albumin being made available to regenerating tissues in proportion to damage and inflammation. In contrast, fibrinogen accumulates at the site of injury in the first 2 weeks, forming fibrin under the influence of enzymes released from the blood and cells surrounding the tissue. Fibrin formation results in an increase in the tensile strength of the wound and leads to the stimulation of fibroblast proliferation and growth.

Phosphorus dendrimers were examined using intrinsic tyrosine fluorescence to see whether they could interact with proteins and change their conformation. The fluorescence spectroscopy method is one of the most popular methods for examining the structure of proteins based on environmental changes in the chromophore environment.

Any changes that occurred in the microenvironment affected the tryptophan residues present in proteins that are responsible for the fluorescence. When a shift in the position of the fluorescence emission maximum occurred, this provided evidence of a change in polarity around the chromophore. A blue-shift of λmax in the HSA emission spectra corresponded to a more hydrophobic location of the amino acid residues and less exposure to the solvent. In contrast, after adding the dendrimers to fibrinogen, there was no significant shift in the maximum emission wavelengths seen, which suggested that no conformational change occurred around the tryptophan residues.^20^ HSA has one tryptophan, so it is easy to observe changes in its environment, while fibrinogen contains a total of 72 intrinsically fluorescent tryptophan residues distributed along the molecule.^57,58^ For this reason, it was difficult to determine whether there were any changes around the chromophore in fibrinogen, as fibrinogen has many tryptophan residues, and a change in the environment around one or several tryptophan residues can be compensated by the fluorescence of other tryptophan residues. The obtained results also indicated a gradual decrease (quenching) of tryptophan fluorescence by cationic phosphorus dendrimers. However, the fluorescence was not completely quenched, meaning that the dendrimers used led to conformational changes in the protein molecule. Research by Klajnert et al. showed that the interaction between protein and dendrimers strongly depended on their surface groups and was greatest in the case of dendrimers ending with a positively charged group (especially the amino group). They suggested that this interaction is electrostatic in nature.^22,59^

The effect of phosphorus dendrimers on the secondary structure of proteins was assessed by using the circular dichroism method. The CD spectra of HSA and fibrinogen were typical of the helical structure. A slight reduction in the intensity of the negative signal was observed, but the similar shape of the CD spectra in the presence and absence of dendrimers indicated that the secondary structure of the tested proteins remained unchanged.

Based on the research, it can be assumed that those slight changes that occurred in the protein conformation did not disturb their physiological functions, as confirmed by results that showed that dendrimers did not affect the ability to form clots.

Examinations of the DPP and DPH dendrimer’s biocompatibility with the hemostatic properties of blood plasma included both an evaluation of the effects on the plasma coagulation cascade and its physiological control mechanism, *i.e.*, fibrinolysis. Analyses of the procoagulant activity of blood plasma in the presence of the dendrimers were based on blood clotting times, which are widely used as diagnostic parameters. Our results showed that phosphorus dendrimers within the range of tested concentrations did not affect the prothrombin and thrombin time significantly, and at low concentrations, they did not cause any changes in the activated partial thromboplastin time either. However, at higher concentrations, they prolonged the activated partial thromboplastin time, and in the case of the DPP dendrimer at the highest concentration used, a complete inhibition of the blood coagulation process was observed which in clinical conditions would lead to bleeding. The tested dendrimers at low concentrations were compatible with blood and did not disturb the coagulation process. The application of TT, PT, and aPTT tests in our study enabled assessing the examined dendrimers effects on two diverse pathways of the blood coagulation cascade activation, *i.e.*, the extrinsic (the tissue factor-dependent) and intrinsic (the contact factor-dependent) coagulation pathway. Both mechanisms occur *in vivo*, but their physiological role and contribution to the fibrin clot formation vary significantly.^60,61^ While the extrinsic pathway is the main physiological hemostatic mechanism during the response to vascular wall injury, the intrinsic pathway acts as a supportive coagulation pathway, and its activation is also involved in the immune response, cooperation with components of the fibrinolytic system, and the tissue remodeling. Moreover, augmented activation of the intrinsic pathway is considered an important mediator of inflammation and thrombotic complications.^62−64^

The PT (prothrombin time) reflects the efficiency of the blood coagulation process dependent on interactions of the tissue factor (a cell membrane protein) with the coagulation factor VII, resulting in an activation of the VII proenzyme into the VIIa enzyme. The TT (thrombin time) parameter provides data on the executive phase of the blood coagulation, *i.e.*, the efficiency of the thrombin-dependent formation of a fibrin clot, which is a final step of the common pathway. The aPTT (the activated partial thromboplastin time) provides information on the efficiency of the intrinsic (the contact factors-dependent) blood plasma coagulation pathway.^65^ This pathway of coagulation is characterized by a different mechanism of activation. It is triggered by a self-activation of coagulation factor XII on negatively charged surfaces (physiological or artificial). Exemplary physiological surfaces for factor XII activation may be collagen exposed in the injured tissues, chromatin networks released by the activated neutrophils, and fragments of the damaged cells. Besides the factor XII, this pathway includes the high molecular weight kininogen (HMWK) and prekallikrein, whose presence is required to effectively activate the factor XI.^66^

Hence, different biochemical mechanisms that are responsible for the initiation of plasma coagulation may result in diversity in the effects of the examined dendrimers on the TT, PT, and APTT. Both the examined dendrimers did not affect the PT and TT, suggesting that they are able neither to interact with serine proteases of the extrinsic pathway nor interrupt the thrombin-catalyzed fibrinogen polymerization. However, the examined dendrimers significantly inhibited the intrinsic pathway of blood plasma coagulation (the aPTT parameter). In this mechanism, the presence of the negatively charged surface and interaction of factor XII with this surface are critical for the initiation of the coagulation process. Under physiological conditions (*i.e.*, in blood plasma), both DPP and DPH are positively charged. Thus, it is likely that the dendrimers may interact with the activation surface, blocking the factor XII activation or interrupting the formation of the contact factors complex (*i.e.*, FXII and its cofactors: prekallikrein and HMWK).

Suty et al. tested the effect of amphiphilic phosphorus dendrons with azacyclopentane groups on blood components. They found that only the combination of a higher concentration and higher dendron generation significantly shortened the thrombin time. However, as in our studies, they also observed an increase in activated partial thromboplastin time when higher concentrations of dendrons were used. They suggested that the observed increase in APTT time was related to the increasing number of available positively charged groups. Abnormalities in routine coagulation parameters depended on the size of the nanoparticles, their concentration, and the number of functional groups. This was in alignment with the higher toxicity observed for amine-modified cationic nanoparticles.^67^

For further investigation of the dendrimer effects on the hemostatic properties of blood plasma, measurements of the blood clotting times were supported by the CCF assay. Due to the presence in the reagent mixture of both thrombin (an executive protease of the coagulation cascade) and t-PA (the main activator of the intravascular fibrinolysis), the CFF assay mimicked a physiological coexistence of blood plasma coagulation and fibrinolytic activity that is necessary to control the size of a fibrin clot, blood fluidity, and hemostatic balance. Considering the results of blood clotting times and the CFF assay, the obtained data indicated that the tested compounds did not affect the extrinsic blood coagulation pathway and fibrinolysis but in higher concentrations could display some reactivity (*i.e.*, anticoagulant properties) toward components of the intrinsic blood coagulation pathway.

Due to the increase in multidrug-resistant organisms in recent years, the search for alternatives to antibiotics has been important. Various groups of dendrimers have been studied for their use as antibacterial agents.^68−72^*S. aureus* is one of the most prevalent bacterial species identified in chronic wounds and skin infections, and the analysis of newly synthesized compounds against this infection is an urgent medical need. Many chronic wounds and ulcers do not heal well, get infected, can develop into gangrene, and possibly end up with amputation. In this context, the antimicrobial potential of 2 phosphorus dendrimers was studied. The DPP dendrimer showed the highest antimicrobial activity against *staphylococcal* strains. Both analyzed compounds had good antimicrobial potential against the *S. aureus* ATCC 6538 strain. Satisfactory results for *E. coli* were also obtained, but among the Gram-negative strains, *P. aeruginosa* was the most resistant to the tested dendrimers. Among the tested compounds, the DPH dendrimer showed the highest activity against the tested microorganisms at higher concentrations. Disruption of anionic bacterial cell membranes is driven through electrostatic interactions.^73^ The differences in the composition of Gram-positive and Gram-negative bacteria cell walls cause distinct resistance to antimicrobial agents. Gram-positive bacteria possess a thick (20–80 nm) cell wall as the outer shell of the cell. In contrast, Gram-negative bacteria have a relatively thin (<10 nm) layer of cell wall but harbor an additional outer membrane with several pores and appendices.^74^ The relatively low permeability of the outer membrane of Gram-negative microorganisms plays a key role in the defense strategy of bacteria since it slows down the penetration of many substances. In our studies, we noted a lower sensitivity of Gram-negative microorganisms to the action of the tested compounds. Moreover, the results indicated that macromolecules with pyridine groups are a more potent antimicrobial agents of both dendrimers.

Apartsin et al. demonstrated that low-generation cationic phosphorus dendrimers expressed concentration-dependent antimicrobial activity toward *S. aureus* and *Enterococcus sp.* Moreover, the dendrimer demonstrated a broad selectivity index against eukaryotic cells.^75^ The viologen-phosphorus dendrimers were also examined for their antibacterial activity against Gram-positive bacteria (*S. aureus* ATCC 6538) and Gram-negative bacteria (*E. coli* ATCC 25922, *Proteus vulgaris* ATCC 13315, and *P. aeruginosa* ATCC 15442).^76^ The report showed that viologen-phosphorus dendrimers built from a hexafunctionalized core (P_3_N_3_)(NCH_3_NH_2_)_6_ and decorated on their surface with aldehyde groups had the highest antimicrobial activity against *S. aureus* and *E. coli*.

## Conclusions

Significant biocompatibility with blood and promising antimicrobial properties of phosphorus dendrimers indicated potential for their use for therapeutic dressings. The tested phosphorus dendrimers could be used as a basis for potential modifications to achieve even better results, thereby favorably influencing the multiphasic wound healing process favorably. The conducted research suggested that these tested compounds could be included in further advanced studies focusing on wound healing.
